# Adiposity and grip strength: a Mendelian randomisation study in UK Biobank

**DOI:** 10.1186/s12916-022-02393-2

**Published:** 2022-06-02

**Authors:** Snehal M. Pinto Pereira, Victoria Garfield, Aliki-Eleni Farmaki, David J. Tomlinson, Thomas Norris, Ghazaleh Fatemifar, Spiros Denaxas, Chris Finan, Rachel Cooper

**Affiliations:** 1 Institute of Sport, Exercise and Health, Division of Surgery & Interventional Science, University College London, London, UK; 2 Institute of Cardiovascular Science, University College London, London, UK; 3 Department of Sport and Exercise Sciences, Musculoskeletal Science and Sports Medicine Research Centre, Manchester Metropolitan University, Manchester, UK; 4 Institute of Health Informatics, University College London, London, UK; 5 UCL British Heart Foundation Research Accelerator, London, UK; 6 Department of Cardiology, Division Heart and Lungs, University Medical Centre Utrecht, Utrecht, Netherlands; 7 AGE Research Group, Faculty of Medical Sciences, Translational and Clinical Research Institute, Newcastle University, Newcastle upon Tyne, UK; 8 NIHR Newcastle Biomedical Research Centre, Newcastle University and Newcastle Upon Tyne Hospitals NHS Foundation Trust, Newcastle upon Tyne, UK

## Abstract

**Background:**

Muscle weakness, which increases in prevalence with age, is a major public health concern. Grip strength is commonly used to identify weakness and an improved understanding of its determinants is required. We aimed to investigate if total and central adiposity are causally associated with grip strength.

**Methods:**

Up to 470,786 UK Biobank participants, aged 38–73 years, with baseline data on four adiposity indicators (body mass index (BMI), body fat percentage (BF%), waist circumference (WC) and waist-hip-ratio (WHR)) and maximum grip strength were included. We examined sex-specific associations between each adiposity indicator and grip strength. We explored whether associations varied by age, by examining age-stratified associations (< 50 years, 50–59 years, 60–64 years,65 years +). Using Mendelian randomisation (MR), we estimated the strength of the adiposity–grip strength associations using genetic instruments for each adiposity trait as our exposure.

**Results:**

In males, observed and MR associations were generally consistent: higher BMI and WC were associated with stronger grip; higher BF% and WHR were associated with weaker grip: 1-SD higher BMI was associated with 0.49 kg (95% CI: 0.45 kg, 0.53 kg) stronger grip; 1-SD higher WHR was associated with 0.45 kg (95% CI:0.41 kg, 0.48 kg) weaker grip (covariate adjusted observational analyses). Associations of BMI and WC with grip strength were weaker at older ages: in males aged < 50 years and 65 years + , 1-SD higher BMI was associated with 0.93 kg (95% CI: 0.84 kg, 1.01 kg) and 0.13 kg (95% CI: 0.05 kg, 0.21 kg) stronger grip, respectively. In females, higher BF% was associated with weaker grip and higher WC was associated with stronger grip; other associations were inconsistent.

**Conclusions:**

Using different methods to triangulate evidence, our findings suggest causal links between adiposity and grip strength. Specifically, higher BF% (in both sexes) and WHR (males only) were associated with weaker grip strength.

**Supplementary Information:**

The online version contains supplementary material available at 10.1186/s12916-022-02393-2.

## Background

Muscle weakness is a major public health concern. Its prevalence increases markedly with age, and it is a key component of age-related conditions such as sarcopenia and frailty [1], which are associated with mobility disability and loss of independence. Hence, the burden of muscle weakness is substantial and shared between individuals, their families and society [2–4]. Measures of muscle weakness are strong predictors of functional decline[5] and against the backdrop of an ageing population [6], the disease and disability burden associated with muscle weakness will continue to rise unless effective public health strategies aimed at reducing its occurrence are implemented. Low absolute grip strength, commonly used to identify muscle weakness, predicts fractures, cardiovascular disease and all-cause mortality [7–10]. Grip strength increases to a peak in early adulthood and is maintained through to mid-life and subsequently declines; but within any one age group, considerable between-individual heterogeneity in strength is evident [11]. While both genetic and environmental factors over the life course are associated with grip strength across adulthood [12, 13], an improved understanding of the causal determinants of strength is still required to help develop effective strategies to prevent or delay the onset of muscle weakness.

Like muscle weakness, adiposity (usually assessed in population-based studies by body mass index (BMI), body fat percentage (BF%), waist circumference (WC) and/or waist-hip-ratio (WHR)) is also associated with a myriad of adverse outcomes including functional decline and premature mortality [5, 14–16]. Obesity is currently highly prevalent in all age groups [17], and BMI and BF% tend to increase with age [18, 19]. Thus, when current generations of younger adults reach older ages, they are more likely to have been overweight or obese for longer proportions of their lives [18] and so have a higher body fat mass than contemporaneous older adults. This has potentially concerning implications for a multitude of age-related outcomes including muscle weakness. Therefore, establishing the influence of lifetime adiposity on strength has become increasingly important.

Most studies exploring associations between adiposity and absolute grip strength have been limited to BMI and findings have been inconsistent with studies showing associations of higher BMI with both weaker [20] and stronger [21] absolute grip, associations in men only [22] or no association [23]. Discrepant findings could be due to differences in age at the time of grip strength assessment or variations in birth cohort and/or country of study. Reliance on BMI as the main marker of adiposity could be another contributing factor. Although BMI provides a quick and easy assessment of total adiposity, it does not distinguish between lean and fat mass. This lack of differentiation is a key limitation when examining relationships with characteristics of muscle, such as strength. In such situations, a more appropriate marker of total adiposity could be BF%, but population-based studies examining relationships between BF% and strength are scant [24]. In addition, while markers of total adiposity are valuable, they do not reflect body fat distribution, and central adiposity has been shown to be a stronger predictor of many health outcomes than total adiposity [15, 16]. For example, central adiposity is characterized by a state of chronic low-grade inflammation, which in turn is associated with lower muscle strength [25, 26]. Yet, few studies have related central adiposity (usually assessed by WC and WHR) to grip strength [21, 22, 24]. Moreover, regardless of the measure of adiposity examined, all observational studies relating adiposity to grip strength suffer from unmeasured and/or residual confounding. For example, capturing information on resistance training which influences strength [27] and may influence adiposity [28] is difficult in large scale studies. Thus, in observational studies, it is not always straightforward to quantify the true effects of adiposity on muscle strength.

Given the importance of understanding whether adiposity causally influences muscle strength and the methodological limitations of existing studies, we aimed to establish the role of total (BMI, BF%) and central (WC, WHR) adiposity on grip strength using UK Biobank data. In an observational analysis, we examined associations between the four indicators of adiposity and grip strength cross-sectionally. To help triangulate evidence on the effect of adiposity on grip strength, we also used an alternative analytic approach—Mendelian randomisation (MR)—to estimate the strength of the same associations using genetic instruments for each of the four different adiposity indicators. Due to evidence of sex-differences in BMI-grip strength associations in previous studies [22], and marked sex-differences in grip strength [11], we chose a-priori to run all analyses stratified by sex.

## Methods

**Fig. 1:**
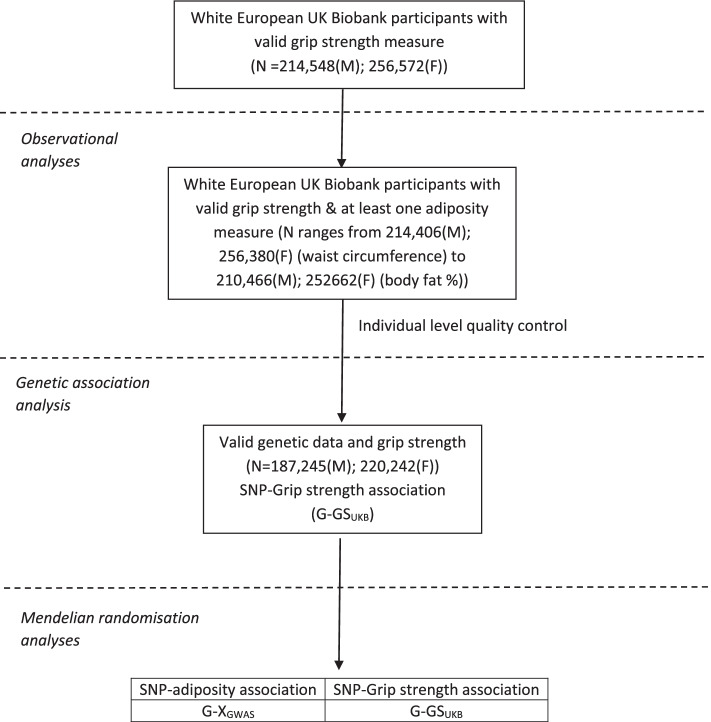
Flow diagram of UK Biobank participants and study design. M, males; F, females; SNP, single-nucleotide polymorphism; G-X_GWAS_, genetic association of adiposity (BMI, BF%, WC and WHR) instruments (SNPs) with BMI, BF%, WC and WHR respectively from relevant GWAS (see methods for details); G-GS_UKB_, genetic association of adiposity instruments (SNPs) with grip strength in UK Biobank

UK Biobank [29, 30] is a prospective study of over 500,000 UK adults aged 38–73 years at recruitment (2006–2010). Participants, recruited across the UK from National Health Service central registers, provided informed consent; ethical approval was given by the North West Multicentre Research Ethics Committee. Our study included only white European participants because the genome-wide association studies (GWAS) used to identify genetic instruments (described below) were primarily based on European samples. The analysis sample of white Europeans with a valid measure of grip strength varied from 470,786 for observational analysis (214,406 males and 256,380 females) to 407,487 for MR analysis (187,245 males and 220,242 females); details are in Fig. 1.

*Adiposity* measures were ascertained following standardised protocols [29]. Weight (without shoes and heavy outer clothing) and BF% were measured using a Tanita BC-418 MA body composition analyser. Height was measured with a Seca 202 height measure; waist and hip circumferences were measured using a tape measure [29]. BMI (kg/m^2^) and WHR were calculated.

*Grip strength* was assessed using a Jamar J00105 hydraulic hand dynamometer. Participants sat upright in a chair with their forearms on armrests. They were asked to squeeze the dynamometer’s handle as hard as they could with their right hand for about 3 seconds. The grip strength measurement was then repeated using the same protocol for the left hand [31]. We examine the maximum recorded value (greater than 0) from either hand.

*Potential confounders* identified a priori, for examined observational associations, are described in detail elsewhere [32]. They included age; deprivation (defined by the Townsend score

[33]); smoking status (never, ex-, current); physical activity (number of days/week participants undertook > 10 min of moderate and vigorous activity, recorded as two separate categorical variables) and average alcohol intake over the last year (rarely/never, once/month to twice/week and ≥ twice/week).

### Adiposity genetic instruments

We used single nucleotide polymorphisms (SNPs) identified from the largest European descendent GWAS to date [34–37], as our genetic instruments for BMI, BF%, WC and WHR (620, 6, 46 and 319 near-independent SNPs, respectively). The SNPs in our genetic instruments were regressed onto the adiposity measure under consideration and the R-squared and F-statistic calculated. Our instruments explained 0.24% (BF%) to 5.81% (BMI) of the variability in adiposity traits in men and 0.17% (BF%) to 5.22% (BMI) in women. The F-statistic varied from 14.90 (WHR) to 73.83 (BF%) in men and from 19.45 (BMI) to 61.52 (BF%) in women (Additional File 1: Table S1). We used genotype dosage information to estimate allele count under an additive genetic model. Instrument details are provided in Additional File 1: Table S2; information on the genetic instrument selection process is provided briefly in Additional File 2: Fig. S1 and in detail elsewhere [38, 39].

## Statistical methods

Due to evidence of sex-differences in BMI–grip strength associations in previous studies [22], and marked sex-differences in grip strength [11], we chose a-priori to run all analyses stratified by sex. Figure 1 illustrates our study design, which includes examination of both observational and genetic associations.

### Correlations between phenotypic and genetic adiposity measures and traits

We calculated pair-wise phenotypic correlations between adiposity measures using Pearson’s correlation coefficients. Genetic correlations between adiposity traits were calculated using cross-trait LD score regressions [40] and GWAS summary statistics [34–37]. The LD score regressions were performed with pre-computed LD scores for each SNP using the 1000 Genomes European data (which are appropriate for use with European GWAS data [40]). We filtered our summary statistics to HapMap3 SNPs, because these are well-imputed in most studies.

### Observational associations

Using linear regression, we examined cross-sectional associations of measured total and central adiposity with grip strength. Analyses were initially minimally adjusted for age (model 1); then additionally adjusted for the potential confounders listed above (model 2). To assess whether associations between adiposity and strength vary by age as has been proposed [41–43], we reran analyses stratified by age groups representing approximate fourths of the population (i.e. < 50 years, 50–59 years, 60–64 years, 65 years +), with tests of interaction between age group and each adiposity measure formally assessed.

### Genetic associations

Briefly, the MR analysis (described below) requires information on associations between individual SNPs (i.e. our genetic instruments, denoted as G) and (i) our outcome, grip strength (denoted as GS) and (ii) our exposure (i.e. the adiposity trait of interest, denoted as X). We calculated individual SNPs genetic association with grip strength (represented by G-GS in Fig. 1) by running linear regressions (with adjustment for 10 genetic principal components); we used individual SNPs genetic association with each adiposity parameter, as reported in the original GWAS [34–37] (represented by G-X). We used a pseudo-two sample MR design, as we had some overlap between the discovery GWAS (for BMI and WHR) and our analytic sample (see the ‘Sensitivity analyses’ section).

### MR analysis

We examined associations between adiposity and grip strength using four different MR approaches (three described here; one described in the ‘Sensitivity analyses’ section). Our main MR model, inverse-variance weighted MR (MR-IVW), estimates the effect of adiposity on grip strength by averaging the genetic instruments’ ratio of instrument–grip strength (G-GS) to instrument–adiposity (G-X) association estimates under a multiplicative random-effects meta-analysis, where the weight of each ratio was the inverse of the variance of the SNP–grip strength association [44, 45]. For the MR-IVW analysis, we report a measure of heterogeneity (I-squared statistic), as heterogeneity could indicate the presence of SNP outliers (which we investigated in the ‘Sensitivity analyses’ section). Our two additional MR analyses, MR-Egger [46] and weighted median MR (MR-WM) [47], allow for horizontal pleiotropy [48] (i.e. they allow for the effects of the genetic instruments on grip strength to *not* be exclusively via their effect on adiposity). MR-Egger produces an intercept term indicative of horizontal pleiotropy [46]; MR-WM gives valid estimates in the presence of horizontal pleiotropy, provided at least 50% of the information in the analysis comes from SNPs that have no pleiotropic effects [47]. When the MR-Egger intercept indicated pleiotropy (*p* < 0.05), we undertook two further analyses. First, funnel plots were examined to identify outlying SNPs, and second, we performed a leave-one-out MR-Egger analysis. We then reran our analyses removing any identified outliers.

The individual SNPs genetic association with each adiposity parameter (G-X) reported in the GWAS were scaled according to a standard deviation increment (SD_X_) of the adiposity trait in the discovery study. Hence, the MR analysis results in effects on grip strength of a one SD increase in the adiposity trait (according to the discovery sample). To ensure resulting coefficients are comparable, observational associations were scaled according to one SD of the adiposity measure of interest.

### Sensitivity analyses

The GWAS from which we identified our BMI and WHR genetic instruments included UK Biobank participants: approximate sample overlap was 60% for BMI and 70% for WHR [36, 37]. We therefore needed to mitigate against over-estimating genetic effect sizes (i.e. winner’s curse bias [49]). We did this by calculating the expected bias and type 1 error rate, using a formula described elsewhere [49]. The expected bias is a linear function of the proportion of overlap between the samples and was calculated given the *F* parameter (the expected value of the *F* statistic), the observational estimate, the sample size and the sample overlap percentage (which we overestimated at 100% for both BMI and WHR). To assess the plausibility of assumptions that underlie our MR analysis, we examined associations between SNPs and potential confounders (Townsend score, smoking status, moderate and vigorous physical activity, and average alcohol intake) using linear/logistic regression as appropriate. Where associations were present, after adjusting for multiple testing using a Bonferroni correction (equating to *p* < 0.05), the MR analysis was re-run excluding potentially invalid SNPs. Finally, to evaluate the MR results by identifying and correcting for potential outliers (*p* < 0.05), we conducted a Mendelian Randomisation Pleiotropy RESidual Sum and Outlier analysis (by running the R function MR-PRESSO [50], with 50,000 bootstrap replications). This method evaluates genetic pleiotropy in the MR model, performs outlier removal, and performs MR again without the outliers to reduce bias in the MR estimates.

We used STATA 14, R, PLINK 2.0 and the command line tool ldsc [40] for data processing and statistical analyses. MR analyses were performed using the *mrrobust* package in STATA [45] and MR-PRESSO function in R [50].

### Data availability

This work was conducted using the UK Biobank resource under application number 71702.

## Results

Mean maximum grip strength was higher in males (41.8 kg; SD = 8.9 kg) than females (25.1 kg; SD = 6.4 kg). Likewise, mean BMI, WC and WHR were higher in males than females, whereas females had higher mean BF% (Table 1).

**Table 1:** Characteristics (Mean(SD)/*N*(%)) of included UK Biobank study participants

	Total	Males	Females
*Outcome*			
Grip strength (kg)^a^	32.7 (11.3)	41.8 (8.9)	25.1 (6.4)
*Exposures*			
BMI (kg/m^2^)	27.4 (4.77)	27.9 (4.24)	27.0 (5.14)
Body fat (%)	31.4 (8.53)	25.3 (5.82)	36.5 (6.89)
WC (cm)	90.3 (13.5)	97.1 (11.4)	84.6 (12.5)
WHR	0.87 (0.09)	0.94 (0.07)	0.82 (0.07)
*Potential confounders*			
Age (years)^b^	56.8 (38–73)	57.0 (38–73)	56.6 (39–71)
Townsend deprivation index^d^	− 1.45 (3.00)	− 1.41 (3.06)	− 1.48 (2.95)
Smoking			
Never	309,446 (65.8)	127,560 (59.6)	181,886 (71.0)
Previous	111,652 (23.7)	60,405 (28.2)	51,247 (20.0)
Current	49,227 (10.5)	26,242 (12.3)	22,985 (8.97)
Days/week moderately active^c^	3.63 (2.33)	3.61 (2.33)	3.64 (2.34)
Days/week vigorously active^c^	1.83 (1.95)	2.04 (2.05)	1.65 (1.85)
Alcohol intake			
Rarely/never	82,951 (17.6)	25,424 (11.9)	57,527 (22.4)
1/month to 2/week	176,554 (37.5)	75,200 (35.1)	101,354 (39.5)
> 2/week	211,275 (44.9)	113,753 (53.1)	97,522 (38.0)

^a^Maximum of left and right hand measures

^b^Range

^c^For more than 10 minutes

^d^Positive values indicate areas with high deprivation; negative values indicate relative affluence

### Correlations between phenotypic and genetic adiposity measures and traits

Phenotypic and genetic correlations were broadly similar. For both phenotypic and genetic correlations and in both sexes, BMI and WC were highly correlated (phenotypic correlation coefficients = 0.88 for both sexes; genetic correlation coefficients = 0.94 (males)/0.90 (females), Additional File 1: Table S3). In females the BMI–WHR and BF%–WHR correlations were low (phenotypic correlation coefficients = 0.46 for both; genetic correlation coefficients = 0.46 and 0.36 respectively), while in males, these correlations were moderate (phenotypic correlation coefficients = 0.60 and 0.63 respectively; genetic correlation coefficients = 0.69 and 0.78 respectively).

### Observational associations: measured adiposity and grip strength

**Fig. 2:**
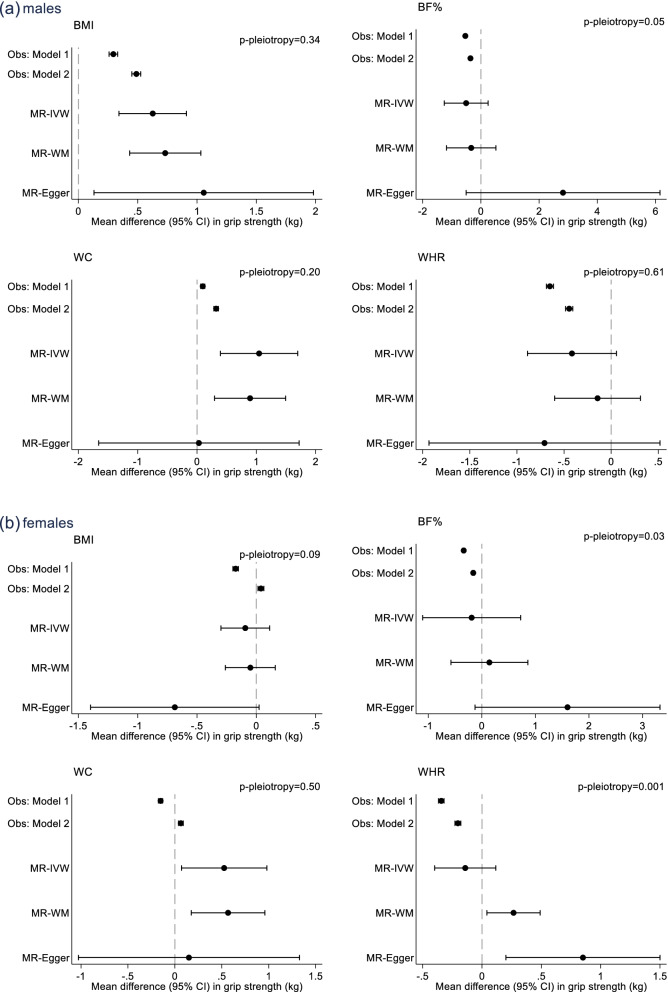
Associations between one SD increases in measured and genetically predicted adiposity (BMI, BF%, WC and WHR) and grip strength in **a** males and **b** females. Numbers represent estimated mean difference in grip strength (kg) per 1 standard deviation higher (measured/genetically predicted) adiposity; p-pleiotropy: *p*-value for overall horizontal pleiotropic effect indicated by the intercept from MR-Egger regression; Obs: model 1 adjusted for age; Obs: model 2 additionally adjusts for Townsend deprivation, smoking, moderate and vigorous physical activity and alcohol intake; MR-IVW, Mendelian randomisation, inverse-variance-weighted; MR-WM, Mendelian randomisation, weighted median estimator; MR-Egger, Mendelian randomisation, Egger; SD, standard deviation; BMI, body mass index; BF%, body fat percentage; WC, waist circumference; WHR, waist hip ratio

**Fig. 3:**
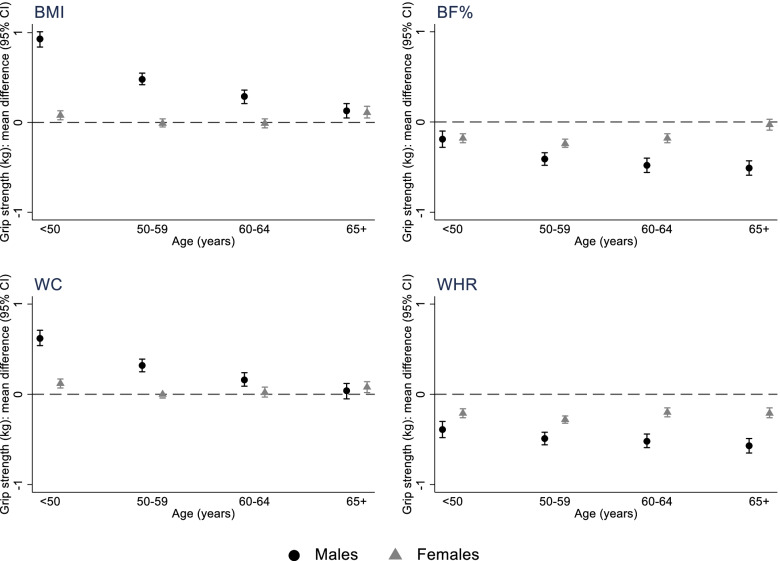
Associations between one SD increases in measured adiposity (BMI, BF%, WC and WHR) and grip strength, stratified by age and sex. Associations adjusted for Townsend deprivation, smoking, moderate and vigorous physical activity and alcohol intake

In males, higher BMI and WC were associated with stronger grip, while higher BF% and WHR were associated with weaker grip (Fig. 2, Additional File 1: Table S4). For example, after adjustment for all covariates, a 1-SD higher BMI was associated with 0.49 kg (0.45 kg, 0.53 kg) stronger grip, while a 1-SD higher WHR was associated with 0.45 kg (0.41 kg, 0.48 kg) weaker grip. With the exception of WHR (*p*_interaction_ = 0.13), there was evidence that associations between adiposity and grip strength varied with age (*p*_interaction_ < 0.001). Relationships of BMI and WC with strength reduced with increasing age, while BF% associations strengthened with age (Fig. 3, Additional File 1: Table S5). For example, in males aged < 50 years, a 1-SD higher BMI was associated with 0.93 kg (0.84 kg, 1.01 kg) stronger grip, whereas among males aged 65 years + , the equivalent estimate was 0.13 kg (0.05 kg, 0.21 kg). Analogous estimates for a 1-SD higher BF% were 0.19 kg (0.10 kg, 0.28 kg) and 0.51 kg (0.43 kg, 0.59 kg) weaker grip for males aged < 50 years and 65 years + respectively.

In females, higher BMI and WC were associated with stronger grip strength, while higher BF% and WHR were associated with weaker grip (model 2; Fig. 2, Additional File 1: Table S4). For example, after adjustment for all covariates, a 1-SD higher WHR was associated with 0.20 kg (0.18 kg, 0.23 kg) weaker grip. There was evidence that all adiposity–grip strength associations varied with age (*p*_interaction_ < 0.01), although the magnitude of differences in associations by age were small (Fig. 3, Additional File 1: Table S5). For example, while there was no association between BMI and grip strength among females aged 50–59 years and 60–64 years, a 1-SD higher BMI was associated with 0.08 kg (0.03 kg, 0.13 kg) and 0.11 kg (0.05 kg, 0.18 kg) stronger grip among females aged < 50 years and 65 + years respectively.

### MR associations between genetically predicted adiposity and grip strength

In males, with one exception (MR-Egger for BF%), all three sets of MR associations were directionally consistent with observed associations. For example, a 1-SD higher BMI was associated with 0.63 kg(0.34 kg, 0.91 kg) stronger grip (MR-IVW; Fig. 2, Additional File 1: Table S4). There was borderline evidence of horizontal pleiotropy for BF% (MR-Egger p-intercept = 0.05). Funnel plots and leave-one-out analyses identified three potentially pleiotropic SNPs (rs1558902, rs6738627 and rs9906944). When BF% analyses were re-run without these SNPs, all three sets of MR associations were directionally consistent with observed associations (Additional File 1: Table S6).

In females, directional consistency with observed associations were present for all three sets of MR associations for WC. In contrast to observed associations, all three sets of MR associations indicated that higher BMI was associated with weaker grip. For BF%, there was evidence of pleiotropy (MR-Egger p-intercept = 0.03), and when potentially pleiotropic SNPs were removed (rs1558902, rs6738627 and rs2943652), two MR associations (MR-IVW and MR-WM) were similar to observed associations, indicating higher BF% was associated with weaker grip (Additional File 1: Table S6). There was also evidence of horizontal pleiotropy for WHR (MR-Egger p-intercept = 0.001). However, both MR-WM and MR-Egger analyses demonstrated directionally consistent associations with grip strength. For example, a 1-SD higher WHR was associated with 0.27 kg (0.04 kg, 0.49 kg) stronger grip (MR-WM). When we excluded identified outlying SNPs (rs1406948, rs10132280, rs12774134), the MR-Egger intercept still indicated pleiotropy (*p* = 0.002); however, both the MR-Egger (slope) and MR-WM associations were similar to those reported in Fig. 2 (see Additional File 1: Table S4 and S6); removing one SNP at a time had little effect on the MR-Egger slope (range: 0.70–0.95) or constant (*p* ≤ 0.005 in all cases).

Any potential bias in our results due to sample overlap (i.e. because the GWAS from which we identified our BMI and WHR genetic instruments included UK Biobank participants) was small: over-estimating sample overlap at 100%, the absolute value of bias was less than 0.01 for both BMI and WHR, with type-1 error rate ≤ 0.06 (Additional File 1: Table S7). We found several associations between SNPs and potential confounders; however, when these SNPs were removed from analyses, results were similar to those presented in Fig. 2 (see Additional File 1: Table S4). There was evidence of heterogeneity for all associations (*I*^2^ = 29.8% to 75.6% (males); 66.0% to 78.7% (females); Additional File 1: Table S4) and MR-PRESSO provided evidence for outliers in all but one case (Additional File 1: Table S8). However, after outlier-correction, there was little change in the strength of evidence of associations.

## Discussion

We investigated evidence for links between different adiposity measures and grip strength in UK Biobank using several complementary approaches and found important sex-differences in associations. In males, our observational analyses indicated that higher BMI and WC were associated with stronger grip while higher BF% and WHR were associated with weaker grip. MR findings were generally directionally consistent with observational findings, providing evidence to suggest observed associations in males are causal. In females, our observational analyses indicated that higher BF% and WHR were associated with weaker grip, while higher BMI and WC were associated with stronger grip. In general, MR associations were only directionally consistent with observed associations for BF% and WC. Finally, our observational analyses suggest that adiposity–grip strength associations could vary by age, which may partly explain some observed inconsistencies.

Our study has several strengths. First, by using MR, we exploit the fact that adiposity alleles inherited by offspring from their parents are randomly distributed, ensuring our MR-associations are less subject to confounding and reverse causation [51]. Second, we adopted four different MR approaches which have distinct strengths and assumptions. The general concordance of results from these different MR approaches with each other and with our observational estimates (in males, and for BF% and WC in females) reinforces the conclusions drawn. Third, by utilising UK Biobank’s large sample size and including multiple genetic instruments, we increased the power of our MR analysis, mitigating against weak instrument bias [52, 53]. Fourth, pleiotropic effects were minimised through MR-Egger [46], MR-WM [47] and MR-PRESSO [50] analyses, and where required (i.e. in males for BF% and in females for BF% and WHR), we explored the effect of individual SNPs. Finally, we attempted to quantify the bias due to overlapping samples for our BMI and WHR analyses, by over-estimating sample overlap to 100%, and found the potential bias in our analyses to be small.

While MR has noteworthy advantages it depends on three main assumptions, the plausibility of which need to be assessed: (i) genetic instruments must associate with the different adiposity traits, (ii) genetic instruments must be independent of potential confounders of the adiposity–grip strength association and (iii) genetic instruments must affect grip strength only via their effect on adiposity (i.e. no horizontal pleiotropy). The first assumption is plausible because the SNPs used as instruments for our adiposity traits were identified from large GWAS [34–37]. While the second and third assumptions are harder to assess definitively, only a relatively small number of SNPs were associated with potential confounders and when we reran our MR analyses without these SNPs, results were similar. However, we acknowledge the possibility that there may be other unmeasured confounders, such as dietary patterns, which may explain the observed pleiotropic WHR associations in females. The assumption of no horizontal pleiotropy is particularly relevant for IVW-MR, because effect estimates can be biased in the presence of unbalanced (directional) horizontal pleiotropy [54]. Fortunately, pleiotropy was observed in only a few instances, and when analyses were rerun removing potentially pleiotropic SNPs, in all but one case (i.e. WHR associations in females), there was no more evidence of pleiotropy. Moreover, the other MR approaches we applied are robust to/correct for horizontal pleiotropy (i.e. MR-Egger [46], MR-WM [47] and MR-PRESSO [50]). Finally, all our MR analyses assume a linear dose–response relationship between adiposity traits and grip strength which may not provide an accurate picture of the shape of the relationship.

Other important methodological considerations include that our observational associations could be affected by measurement error. For example, BF% was estimated by bio-impedance, the accuracy of which depends on several factors [55]. Nonetheless, bio-impedance is a valid estimation technique for large epidemiological studies [56], such as UK Biobank. Rather than examining a single adiposity indicator (BMI) as in most previous work, we consider a range of measures including a measure of total adiposity that distinguishes fat from lean mass (BF%) and indicators of central adiposity (WC and WHR). Nevertheless, other novel adiposity measures such as visceral adipose tissue [57], ‘favourable’ and ‘unfavourable’ adiposity [58] were not considered and warrant investigation in future research. We were limited to using grip strength as our marker of muscle strength. Although grip strength is a convenient and commonly used proxy for overall body strength and the Jamar dynamometer used has good reliability and reproducibility [59], grip strength specifically measures upper limb strength. Evidence on whether grip strength is an adequate proxy for overall muscle strength is equivocal [60, 61] and relationships between adiposity and strength are likely to vary by location of strength measurement (e.g. loaded vs. unloaded muscle). In terms of generalizability of findings, our analysis was restricted to white European participants and thus we are unable to extrapolate to other ethnic groups. Additionally, patterns of selection into studies such as UK Biobank (i.e. “healthy volunteers” [62]) can induce collider bias. Even modest influences on selection could lead to biased estimates [63], and we acknowledge that such biases can affect both observational and instrumental variable analyses [64]. Therefore, future work is needed to assess whether our findings are replicated in other samples including those representing other ethnic groups.

Our findings for total and central adiposity in males broadly agree, in terms of direction of associations, with other studies [22]. Findings for females, both within our study and more generally, compared with the literature, are less consistent. For example, in observational analyses for females, we found higher BMI was associated with stronger grip, contrasting with a meta-analysis that found no association [22]. In observational analyses, for males, we found 0.12 kg stronger grip for a 1 kg/m^2^ higher BMI (i.e. mean difference in grip strength per BMI-SD (0.489)/BMI-SD (4.24)). This concurs with findings from a meta-analysis that in males (mean age ranging from 53 to 79 years), a 1 kg/m^2^ higher BMI was associated with 0.23 kg stronger grip [22]. Similar to us, the meta-analysis noted associations in males between BMI and grip were stronger at younger ages. While our MR analyses estimates the life-long effect of BMI on strength, it provides little information regarding the importance of BMI at specific life-stages. The importance of BMI at specific life-stages for strength is supported in the literature in terms of both empirical evidence and biological plausibility. For example, cross-sectional analyses of age-heterogeneous samples suggests that relationships between adiposity and muscle characteristics vary by age: muscle adapts beneficially to loading induced by adiposity at younger ages, but this benefit is not seen at older ages [41–43]. These age-related differences noted elsewhere [41–43] concur with our observations and need replication in cohorts of individuals with detailed repeat measures of both adiposity and strength over time. This is essential to understand how lifetime adiposity influences strength at specific life-stages and changes in strength over life. Underlying biological mechanisms explaining the changing relationship between adiposity and strength with age, including observations that with advancing age, chronic low-grade inflammation, insulin resistance and hormone dysregulation due to long-term exposure to obesity [65], could eventually override the beneficial anabolic stimulus to muscle provided by loading from adiposity in earlier adulthood. Future research should explore these underlying mechanisms with the adiposity measures examined here and other phenotypes such as ‘favourable’ and ‘unfavourable’ adiposity [58], which, although associated differentially with metabolic profiles, are similarly associated with inflammatory markers such as CRP [58].

Unlike BMI, BF% provides an indicator of total adiposity that distinguishes fat from lean mass, and reassuringly, higher BF% in both sexes was consistently associated with lower grip. This finding is important in highlighting the need to examine different dimensions of adiposity. Both BMI and BF% are markers of total adiposity and are correlated; however, the direction of association with strength varied for BMI and BF% in males. In females, the MR associations for BMI and BF% both indicated adverse associations with strength. These somewhat unexpected observations could be explained by BMI’s inability to differentiate between lean and fat mass and the observation that females tend to have proportionally more fat mass than males [66]. Therefore, at a population level, BMI in males might be more representative of lean mass and in females more representative of fat mass. In men, our finding that higher WC was related to stronger grip agrees with the meta-analysis [22]. WHR was only moderately correlated to BMI; hence, it is likely to be a more specific surrogate for central fat deposition (compared to WC). We found that in men, higher WHR was consistently associated with lower grip. For example (in observational analysis), for a man in UK Biobank with average hip circumference (104 cm), a 7 cm greater WC was associated with 0.45 kg weaker grip, and although the magnitude of association is small, it is relevant, because a 1 kg reduction in grip strength is associated with a 3% increase in mortality rates [8]. In females, while observational associations between BMI and WC concur, MR associations are in opposite directions, despite high levels of genetic correlation between the two traits. Moreover, our inability to explain pleiotropy in WHR associations for females is also concerning. Thus, our findings regarding associations between adiposity and grip strength in females warrant further investigation. Nonetheless, our findings for BF% in males and females and WHR in males add to the sparse literature to date and are noteworthy because both BF% and abdominal adiposity increase with age [19, 67].

## Conclusions

The importance of maintaining muscle strength at older ages is increasingly being recognised [27]. Population ageing [6], coupled with current [17], and projected [18] obesity trends necessitate that we establish the influence of adiposity on muscle strength. Moreover, because ageing is associated with a progressive loss of subcutaneous fat and the accumulation of visceral and ectopic fat [68], it is important to elucidate how not only markers of total adiposity which distinguish between fat and muscle but also more specific adiposity markers influence strength. While acknowledging that relationships between adiposity and strength may vary with age and could be bi-directional [69], our consistent findings using different methodological approaches strengthens the evidence base for causal links between total adiposity and grip strength in males and females and between central adiposity and grip strength in males. We found that for both sexes, higher BF% and (in males only) WHR was associated with weaker grip strength. Pathways linking total and central adiposity to strength are likely to differ; for example, loci associated with BMI implicate pathways that act in the brain, whereas loci associated with fat distribution point to pathways involved in adipocyte biology and insulin resistance [70]. Further study is warranted to establish underlying mechanisms explaining our findings, before transitioning to clinical trials that can inform on the potential translation of these insights towards benefiting the general population.

## Supplementary Information

**Additional file 1: Tables S1-S8. Table S1.** R-squared and F-statistic of adiposity genetic instruments. **Table S2.** Summary statistics describing SNP associations with (i) adiposity exposures (BMI, BF%, WC and WHR) and (ii) grip strength. **Table S3.** Correlation coefficients between adiposity measures for males and females: (a) phenotypic correlations, (b) genetic correlations. **Table S4.** Mean difference (95% CI) in grip strength (kg) by markers of total and central adiposity. **Table S5.** Mean difference (95% CI) in grip strength (kg) by markers of total and central adiposity, stratified by age. **Table S6.** Mean difference (95% CI) in grip strength (kg) by markers of total and central adiposity: exploring pleiotropy. **Table S7.** Estimation of bias due to sample overlap. **Table S8.** MR PRESSO Outlier correction.

**Additional file 2: Figure S1.** Genetic instrument selection process.

## Data Availability

The data that support the findings of this study are available from UK Biobank project site, subject to successful registration and application process. Further details can be found at https://www.ukbiobank.ac.uk/.

## Abbreviations

BF%: Body Fat Percentage
BMI: Body Mass Index
GWAS: Genome-Wide Association Studies
MR: Mendelian Randomisation
SD: Standard Deviation
SNP: Single Nucleotide Polymorphism
WC: Waist Circumference
WHR: Waist-Hip-Ratio
