# Supplementary material for: Adiposity and grip strength: a Mendelian randomisation study in UK Biobank
Source: BMC Med. 2022 Jun 2;20:201. doi: 10.1186/s12916-022-02393-2 (PMC9161610; doi:10.1186/s12916-022-02393-2)
Supplement: Supplementary file 2 — Additional file 2: Figure S1. Genetic instrument selection process. [file 12916_2022_2393_MOESM2_ESM.docx]

**Figure S1: Genetic instrument selection process**

BMI

**657 SNPs***

(Yengo, 2018)

BF%

**6 SNPs***

(Lu, 2016)

WC

**46 SNPs***

(Shungin, 2015)

WHR

**370 SNPs***

(Pulit, 2019)

Filtering: Linkage disequilibrium (LD) clumping**

Filtering: Linkage disequilibrium (LD) clumping**

Filtering: Linkage disequilibrium (LD) clumping**

Filtering: Linkage disequilibrium (LD) clumping**

Available for analysis in our sample: **620 SNPs**

*Minor allele frequency (MAF) filtering at 0.01 previously done in the GWASs for each exposure. Genome wide significance in the original GWAS defined as p<1x10^-8^ for BMI, p<5x10^-8^ for BF% and WC and p<5x10^-9^ for WHR **passed genetic QC and confirmed as independent using LD clumping thresholds of r^2^<0.001 and a 250kb window, reference data used was 1000 Genomes CEU.

Available for analysis in our sample: **319 SNPs**

Available for analysis in our sample: **46 SNPs**

Available for analysis in our sample: **6 SNPs**
